# Are women birthing in New South Wales hospitals satisfied with their care?

**DOI:** 10.1186/s13104-015-1067-2

**Published:** 2015-03-28

**Authors:** Jane B Ford, Diane M Hindmarsh, Kim M Browne, Angela L Todd

**Affiliations:** C/- University Department of Obstetrics and Gynaecology, Kolling Institute, University of Sydney, Building 52, Royal North Shore Hospital, St Leonards, Sydney, NSW 2065 Australia; Bureau of Health Information, PO Box 1770, Chatswood, Sydney, NSW 2057 Australia

**Keywords:** Patient survey, Satisfaction with care, Maternity, Patient experience

## Abstract

**Background:**

Surveys of satisfaction with maternity care have been conducted using overnight inpatient surveys and dedicated maternity surveys in a number of Australian settings, however none have been used to report on satisfaction with maternity care among women in New South Wales. The aims of this study were to investigate the association between: 1) parity (first and subsequent births) and patient experience of hospital care at birth, and 2) other patient, birth and hospital characteristics and experience of hospital care at birth.

**Methods:**

Data were from the New South Wales (NSW) Ministry of Health surveys of overnight hospital inpatients, including maternity patients, between 2007 and 2011. Questionnaires were mailed to a sample of patients three months after receiving inpatient services involving at least 1 night in a public hospital. Experience of care included 12 items grouped into: satisfaction with care, staff and information. Results were weighted to overall hospital facility populations and age-standardised. Frequencies and chi-square tests were used.

**Results:**

Analysis of responses from 5,367 obstetric patients revealed three quarters of women were satisfied with care provided in hospital. Compared with women who had previously given birth, first-time mothers were more likely to recommend their birth hospital to friends and family (60.5% versus 56.4%; P < 0.05), less likely to have experienced differing messages from staff (44.8% vs 59.4%; P < 0.001), and less likely to feel they had received sufficient information about feeding (58.8% vs 65.0%; P < 0.001) and caring for their babies (52.4% vs 65.2%; P < 0.001). While metropolitan women were more likely to rate their birth hospital positively (76.0% vs. 71.3%; P < 0.05) than their rural counterparts, rural women tended to rate the care they received (68.1% vs. 63.4%; P < 0.05), and doctors (70.7% vs 61.1%; P < 0.05) and nurses (73.5% vs. 66.9%; P < 0.001) more highly than metropolitan women.

**Conclusions:**

The overall picture of maternity care satisfaction in New South Wales is a positive one, with three quarters of women satisfied with care. Further resources could be dedicated to ensuring consistency and amount of information provided, particularly to first-time mothers.

**Electronic supplementary material:**

The online version of this article (doi:10.1186/s13104-015-1067-2) contains supplementary material, which is available to authorized users.

## Background

In the context of maternity policies with an increasing focus on woman-centred care [1-3], numerous international surveys of women’s satisfaction with hospital maternity care provision have been undertaken [4-10]. Generally, these surveys report high levels of satisfaction with care provided [4-10].

Satisfaction with care is an artificial construct and is likely to be affected by respondent characteristics and study design components [11]. Ideally attempts to measure satisfaction in surveys should involve some effort to validate satisfaction via another outcome measure (e.g. satisfaction with clinical waiting times and walkouts), correlation with other satisfaction measures within the survey and additional qualitative research that can supplement findings [11,12].

A woman’s experience of maternity care is multi-dimensional (staff, hospital, decision-making, information) and measurement is complicated by issues of person, time, place and population [13]. Surveys have considered a number of factors that may influence overall experience of care including parity [4-6], area of residence [6,8], labour and birth characteristics [5], hospital type [6,9], length of stay [8,9], number of caregivers during pregnancy [4], having previously met the midwife providing birth care [4], and interactions with staff. [5] The influence of such factors on overall experience can provide important insights to policy-makers into how women perceive their maternity care and the factors that may improve care.

While targeted maternity satisfaction surveys have been conducted in Australia in Victoria [5] and Queensland [6] and as part of overall patient surveys in South Australia [8] and Western Australia [9], the satisfaction of women receiving maternity care in New South Wales (representing one-third of Australian births) has not been investigated to date. New South Wales patient survey reports have excluded obstetric patients despite collecting responses from these women [14,15]. The aims of this study were to investigate the association between: 1) parity (first and subsequent births) and patient experience of hospital care at birth, and 2) other patient, birth and hospital characteristics and patient experience of hospital care at birth using data from the NSW patient surveys undertaken between 2007 and 2011.

## Methods

New South Wales (NSW) Ministry of Health conducted surveys of overnight hospital inpatients, including maternity patients, between 2007 and 2011. Questionnaires developed specially for NSW Health by Picker/NRC [13] were mailed to a sample of patients who received inpatient services and stayed for at least one night in public hospitals in NSW. The survey design involved a stratified random sample from all facilities offering services during the selected timeframe. Between 2007 and 2009 patients receiving services during a single month (February) were surveyed and between 2010 and 2011 an approximately equal sample was selected from each month of the year. A questionnaire was mailed to each selected patient approximately three months following their receipt of care. Thirteen days later a reminder letter was sent, followed by an additional questionnaire 3 weeks later to those who had not returned a completed questionnaire. At larger facilities, a relatively small proportion of the patient population was selected whereas at smaller facilities the entire population of patients may have been selected. The response rate between 2007 and 2010 was 44%, and in 2011 was 36%. Response rates were not reported by patient care categories (e.g. among obstetric patients). Children under 17 years, newborns, mental health and rehabilitation patients were not eligible for participation in the survey.The data were weighted to the patient population by broad age groups (17–49,50+) within each facility.

While the majority of questions in the NSW overnight hospital inpatient questionnaire were targeted at all male and female inpatients, there were a few specific obstetric questions including: mode of birth, parity (first or subsequent birth) and satisfaction with information provided about caring for and feeding a baby. Obstetric patients for whom responses are presented in this paper were identified as female patients of reproductive age (20–59) attending a public hospital who responded to questions about mode of birth and whether their hospital stay related to a first or subsequent birth (Additional file 1). Responses were restricted to those from hospitals known to provide maternity services.

Experience of care for the purposes of this research was assessed using 12 questions grouped into three aspects of patient care: satisfaction with care in hospital, satisfaction with staff and satisfaction with information provided. *Satisfaction with care* included how patients rated the hospital and the care they received in hospital and whether they would recommend the hospital to friends; *satisfaction with staff* included ratings on courtesy, how well doctors and nurses worked together, whether patients received different messages from doctors and nurses, and whether they perceived their care provider had a full understanding of their condition and treatment; and *satisfaction with information provided* included whether patients received understandable responses from doctors and nurses, and whether they received enough information about feeding and caring for their baby. While there were numerous other patient experience questions in the overnight patient survey, many of these were not applicable to maternity patients. Three scales were used in the 12 questions considered in this paper: a three category scale for questions on specific aspects of care (‘yes completely’, ‘yes somewhat’ coded as positive, ‘no’ as negative and missing), a 0–10 scale for hospital rating (aggregated into negative or neutral (0–6), positive (7–10) and missing)) or a five category scale for other rating-type questions (poor, fair, good, very good, excellent) with very good and excellent combined for positive ratings. Aggregation of ratings is consistent with previous reporting of findings from the overnight patient survey [15]. The original survey was developed and copyrighted by NRC Picker and uses questions that have been tested in multiple settings and shown to have high internal consistency [16]. Analysis of all inpatient responses has indicated that overall ratings of care are related to experiences of staff interactions and responsiveness as well as cleanliness and waiting times [17].

Maternal, pregnancy and birth characteristics included maternal age group, language spoken, parity, self-rated health status and mode of birth as reported by women in the survey. Type of care included whether or not one particular doctor was in charge during the hospital stay. This was used as a proxy for continuity of obstetrician care. Self-rated health status is commonly used in patient surveys and has been reported to be consistent with factors important to health and fitness [18] Rural hospitals were defined as those for which remoteness area classification was not “major city” [19].

This secondary analysis of the data used the existing survey weights based on the overall hospital facility populations, trimmed to avoid excessive weights. Chi-squared tests based on the survey logistic procedure in SAS V9.3 were used to assess significant differences between groups, with the facility and age included as strata. A finite population correction factor was not included as the proportion of the obstetric population included in the survey was small. Results were age-standardised to the overall age distribution according to the 2007 to 2010 age distribution in the Perinatal Data Collection [20]. Ethical approval for this study was provided by the NSW Population and Health Services Research Ethics Committee (2013/07/027).

### Ethics approval

Ethical approval for this study was provided by the NSW Population and Health Services Research Ethics Committee (2013/07/027).

## Results

There were 5,554 (15.5%) women among the 35,797 female population surveyed who indicated they had given birth. Following exclusions for missing responses on mode of delivery (n = 111) there were 5,367 (15.0%) women receiving inpatient obstetric care at 75 hospitals with responses available for analysis.

For 2,412 women (44.9%) this was their first childbirth experience (primiparous) and 2,955 women (55.1%) had previously given birth (multiparous) (Table 1). Compared to multiparous women, primipara were younger, had slightly better self-rated health, and were more likely to be non-English speakers and to be giving birth in a metropolitan hospital. There were no differences between women having first or subsequent births in the proportions of women under the care of one doctor or the proportions of women having a caesarean section (Table 1). Higher proportions of women in rural compared to metropolitan areas reported very good or excellent health and also reported that they experienced one particular doctor in charge.

**Table 1:** **Characteristics of women with first and subsequent births**

**Patient characteristics**	**First birth**	**Subsequent birth**	**Total births**
	**N = 2412**	**N = 2955**	**N = 5367**
	**N (%)**	**N (%)**	**N (%)**
*Mode of birth*			
Vaginal	1720 (71.3)	2075 (70.2)	3795 (70.7)
Caesarean	692 (28.7)	880 (29.8)	1572 (29.3)
*Age group†*			
20-29	1245 (51.6)	925 (31.3)	2170 (40.4)
30-39	1087 (45.1)	1809 (61.2)	2896 (54.0)
40-49	76 (3.2)	213 (7.2)	289 (5.4)
50-59	4 (0.2)	8 (0.3)	12 (0.2)
Language spoken at home*			
English	1935 (80.2)	2465 (83.4)	4400 (82.0)
Non-English	329 (13.6)	309 (10.5)	638 (11.9)
Missing	148 (6.1)	181 (6.1)	329 (6.1)
Hospital location*			
Metropolitan	1631 (64.6)	1737 (58.8)	3368 (62.8)
Rural	781 (32.4)	1218 (41.2)	1999 (37.2)
Year surveyed*			
2007	454 (18.8)	578 (19.6)	1032 (19.2)
2008	485 (20.1)	594 (20.1)	1079 (20.1)
2009	601 (24.9)	732 (24.8)	1333 (24.8)
2010	351 (14.6)	503 (17.0)	854 (15.9)
2011	521 (21.6)	548 (18.5)	1069 (19.9)
*One particular doctor in charge of care in hospital*			
Yes	1163 (48.2)	1475 (49.9)	2638 (49.2)
No/not sure	894 (37.1)	1045 (35.5)	1939 (36.1)
Missing	355 (14.7)	435 (14.7)	791 (14.7)
Self-rated health*			
Poor/fair	52 (2.2)	63 (2.1)	115 (2.1)
Good	383 (15.9)	547 (18.5)	930 (17.3)
Very good/excellent	1962 (81.3)	2319 (78.5)	4281 (79.8)
Missing	15 (0.6)	26 (0.9)	41 (0.8)

Significant differences between women experiencing first and subsequent births are noted as follows: *P < 0.05, †P < 0.001. Note: percentages in this table are unweighted.

**Figure 1:**
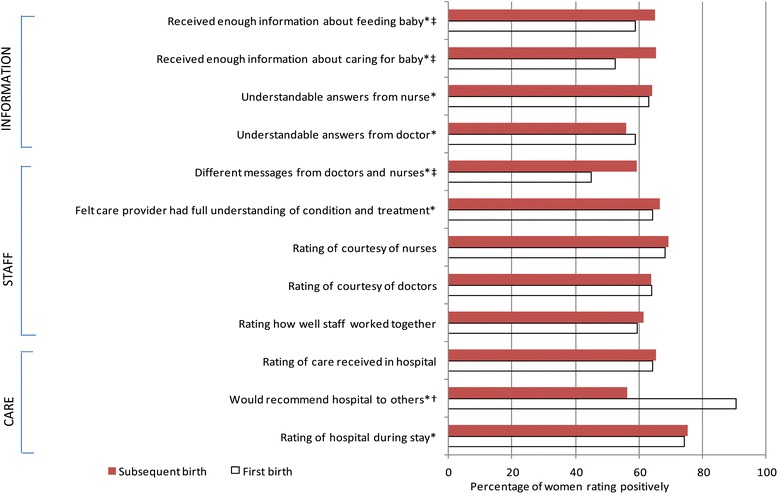
**Satisfaction with care among women experiencing first and subsequent births.** Results have been weighted and age-standardised. Includes positive ratings (denoted with*) or assessment as very good or excellent (all other questions). Significant difference between women experiencing first and subsequent births are highlighted in bold: †P < 0.05, ‡P < 0.001.

Overall, women experiencing a subsequent birth rated their care (on 10 of the 12 items) more highly than first-time mothers. Significant differences between mothers having a first and subsequent birth were evident in 8 of the 12 satisfaction with care items. First-time mothers were more likely to recommend their birth hospital to friends and family, less likely to have experienced differing messages from staff and less likely to feel they had received sufficient information about feeding and caring for their babies, than women who had previously given birth (Figure 1).

### Satisfaction with care

Three quarters (75.3%) of women positively rated the hospital they stayed at (Table 2). While 64.7% of women positively rated the care they received in hospital, 58.4% of women would recommend the hospital to friends and family. Women attending hospitals in metropolitan areas were more likely to positively rate their birth hospital and care received than their rural counterparts. Mode of birth did not affect satisfaction with the care provided. Women with very good or excellent health status were more likely to rate their hospital stay positively as were women who perceived one particular doctor to be in charge of their care in hospital.

**Table 2:** **Were ratings of care among obstetric patients affected by mode of delivery, location, patient health status and continuity of care?**

**Dimension**	**Rating**	**Overall**	**Mode of delivery**		**Hospital location**		**Self rated health status**		**Perceived one doctor in charge of care**	
		**N = 5367**	**Caesarean**	**Vaginal**	**Metropolitan**	**Rural**	**Poor/fair/good**	**Very good/excellent**	**One doctor**	**>1 doctor‡**
			**N = 1572**	**N = 3795**	**N = 3368**	**N = 1999**	**N = 1045**	**N = 4281**	**N = 2638**	**N = 1939**
		**(col %)**	**(col %)**	**N (col %)**	**N (col %)**	**N (col %)**	**N (col %)**	**N (col %)**	**N (col %)**	**N (col %)**
*Rating of hospital during stay*	Positive	74.8	72.8	75.5	76.0*	**71.3**	**63.7†**	**77.9**	**78.0†**	**71.7**
	Negative	24.3	26.6	23.6	**23.0**	**27.5**	**34.8**	**21.3**	**21.3**	**27.3**
	Missing	1.0	1.1	0.9	**0.9**	**1.1**	**1.6**	**0.8**	**0.7**	**1.0**
*Would recommend this hospital to friends and family*	Positive	58.4	57.4	58.7	**60.4†**	**53.1**	**46.7†**	**61.6**	**62.8†**	**54.3**
	Negative	46.7	41.6	40.3	**38.7**	**45.7**	**51.9**	**37.6**	**36.5**	**44.7**
	Missing	1.0	1.0	0.9	**0.9**	**1.2**	**1.5**	**0.8**	**0.7**	**1.0**
*Rating of care received in hospital*	Very good/excellent	64.7	63.6†	65.1	63.4*	**68.1**	**46.2†**	**69.8**	**70.0†**	**60.1**
	Poor/fair/good	34.3	35.4	33.9	**35.6**	**31.1**	**51.9**	**29.5**	**29.3**	**38.8**
	Missing	1.0	1.0	1.0	**1.0**	**0.8**	**1.9**	**0.7**	**0.7**	**1.1**
*Rating how well the doctors and nurses worked together*	Very good/excellent	60.3	58.6	61.0	**58.0†**	**66.1**	**42.3†**	**65.4**	**67.6†**	**54.0**
	Poor/fair/good	38.5	40.1	37.9	**40.6**	**32.9**	**55.4**	**33.7**	**31.6**	**44.6**
	Missing	1.2	1.3	1.2	**1.3**	**0.9**	**2.3**	**0.9**	**0.8**	**1.4**
*Rating of courtesy of doctors*	Very good/excellent	63.8	**69.0†**	**61.7**	**61.1†**	**70.7**	**47.7†**	**68.5**	**76.7†**	**53.1**
	Poor/fair/good	33.7	**29.0**	**35.5**	**36.1**	**27.4**	**47.8**	**29.6**	**22.1**	**43.6**
	Missing	2.5	**2.0**	**2.7**	**2.8**	**1.8**	**4.5**	**1.9**	**1.1**	**3.2**
*Rating of courtesy of nurses*	Very good/excellent	68.7	67.0†	69.4	**66.9†**	**73.5**	**53.1†**	**73.0**	**72.4†**	**64.6**
	Poor/fair/good	29.6	31.2	29.0	**31.3**	**25.2**	**43.9**	**25.7**	**26.4**	**33.6**
	Missing	1.7	1.8	1.6	**1.8**	**1.3**	**3.0**	**1.3**	**1.3**	**1.8**
*Felt care provider had a full understanding of condition and treatment*	Positive	65.4	64.2	65.9	**63.3†**	**71.0**	**52.1†**	**69.0**	**74.4†**	**59.8**
	Negative	33.1	34.3	32.6	**35.1**	**27.8**	**45.5**	**29.8**	**24.7**	**38.5**
	Missing	1.5	1.6	1.5	**1.6**	**1.1**	**2.5**	**1.2**	**0.9**	**1.7**
*Different messages from doctors and nurses*	Positive	52.4	**46.1†**	**54.9**	52.5	52.1	**45.6†**	**54.3**	**55.9†**	**48.7**
	Negative	46.6	**52.7**	**44.3**	46.5	46.9	**53.1**	**44.8**	**43.5**	**50.5**
	Missing	1.0	**1.2**	**0.9**	1.0	1.0	**1.3**	**0.9**	**0.6**	**0.8**
*Understandable answers from doctor*	Positive	57.5	**64.3†**	**54.6**	**55.4†**	**62.8**	52.6*	**58.9**	**74.1†**	**43.6**
	Negative	30.0	**26.1**	**31.7**	**31.2**	**27.0**	**32.6**	**29.3**	**20.6**	**39.3**
	Missing/did not have questions	12.5	**9.6**	**13.7**	**13.4**	**10.2**	**14.8**	**11.9**	**5.3**	**17.2**
*Understandable answers from nurse*	Positive	63.6	62.0	64.2	63.3	64.3	**55.3†**	**66.0**	**67.1†**	**59.7**
	Negative	34.4	35.8	33.9	34.7	33.7	**41.9**	**32.3**	**31.4**	**38.1**
	Missing/did not have questions	2.0	2.1	1.9	2.0	1.9	**2.8**	**1.8**	**1.6**	**2.2**
*Enough information about feeding baby*	Positive	62.1	60.9	62.5	62.6	60.7	56.0*	**63.7**	**65.2†**	**58.6**
	Negative	37.1	38.2	36.6	36.6	38.4	**43.0**	**35.5**	**34.1**	**40.5**
	Missing	0.8	0.9	0.8	0.8	0.9	**1.1**	**0.8**	**0.7**	**0.9**
*Enough information about caring for baby*	Positive	59.1	59.5	59.0	58.3	61.2	53.2*	**60.6**	**64.8†**	**54.3**
	Negative	40.2	39.8	40.3	40.9	38.2	**45.9**	**38.7**	**34.7**	**45.0**
	Missing	0.7	0.7	0.7	0.7	0.7	**0.9**	**0.7**	**0.5**	**0.8**

Percentages are age-standardised and weighted; Percentages may not add to 100% due to rounding. Significant differences are highlighted in bold: *P < 0.05, †P < 0.001. Differences are for each variable compared to the cell to the right. ‡ It may also be that women perceive no doctor is in charge.

### Satisfaction with staff

When compared to women birthing in metropolitan hospitals, rural women were more likely to feel their care provider had a full understanding of their condition and treatment, and perceive that the doctors and nurses worked well together (Table 2). Women birthing in rural hospitals were also more likely to rate the courtesy of doctors and nurses positively. Women who perceived there was one doctor in charge of their stay were more likely to rate all aspects of staff care provision positively when compared to women who did not perceive one doctor was in charge.

Women’s health status also affected satisfaction with staff. Women rating their health status as very good or excellent were more positive about all aspects of care provided by staff than women with poor, fair or good health status (Table 2). Mode of delivery only affected a few aspects of satisfaction with staff – women having a caesarean birth were more likely to rate the courtesy of doctors as very good or excellent and more likely to have a negative experience of differing messages from doctors and nurses than women giving birth vaginally.

### Satisfaction with information

Mode of delivery, geographical location, self-rated health status and perception of doctor in charge all affected whether women felt they received understandable *information from doctors*, with more positive ratings among women having a caesarean birth, in a rural hospital, with very good or excellent health status and/ or with one doctor perceived to be in charge. Similar patterns were evident in relation to *information from nursing staff*, although the only significant differences in responses were related to health status and perceived doctor in charge. A higher proportion of women overall felt they received understandable answers from nurses (63.6%) than doctors (57.5%) when they had important questions to ask (Table 2).

Overall, 62.1% of women felt they had sufficient information about feeding their baby while 59.1% of women felt they received sufficient information about caring for their baby (Table 2). Women with very good or excellent health status and the perception of one doctor in charge were the most likely to positively rate having had sufficient information about feeding and caring for their baby.

There were few changes in ratings when the two cohorts (2007–09, 2010–11) were compared. The proportion of women perceiving one doctor to be in charge of their care increased slightly over time (4%). The earlier cohort were slightly more likely to rate nurse/doctor teamwork positively and feel their care provider had a full understanding of their condition and treatment. However, increases were marginal (data not shown).

## Discussion

Overall, three quarters of women were satisfied with care provided in hospital. We found significant differences in women’s ratings of some aspects of care, staff and information provided. First-time mothers were more likely to recommend their birth hospital to friends and family, more likely to have experienced consistent messages from staff and less likely feel they had received sufficient information about feeding and caring for their babies than women who had previously given birth.

Overall rates of satisfaction were slightly lower than those reported in the UK [7] (87% of women were satisfied or very satisfied), but consistent with those reported in a Queensland survey where 71% of women reported being cared for ‘very well’ during labour and birth [6]. Consistent with other surveys [6,7], women with previous experience of giving birth were more likely to be positive about their care. It has been suggested that when women are rating their overall care, satisfaction is likely to be driven by experiences of postnatal rather than antenatal or care at birth [4].

It is difficult to compare satisfaction ratings across international and national settings, given the use of different rating scales. There is some evidence to suggest that there are differences in perceptions of patients who are ‘highly satisfied’ compared to ‘satisfied’, with only the former group perceiving optimal care [21]. However, we had a limited opportunity to explore sub-categories given reliance on pre-specified aggregation of responses and sample size restrictions. Clearly, satisfaction is a complex concept that is difficult to explore in depth using questionnaires, particularly when there is no opportunity to separate care across different aspects of hospital stay. Dedicated maternity surveys are able to separate women’s satisfaction with labour and birth care from postnatal care which is not possible in general patient surveys. However, comparison of satisfaction among different subgroups of the maternity population (by parity, mode of delivery and geography for example) can provide insight into relative satisfaction. Overall maternity patients in Australia, Canada and the UK report consistently high levels of satisfaction with maternity care: proportions of satisfied women are above 65% [4-7,10] and satisfaction scores above 80% [8,9]. Further research untangling the attitudes and expectations, issues of control and well-being, relationships, and individualized care related to satisfaction scores in each of these settings would be worthwhile [12].

A particularly interesting finding was the increased likelihood of first-time mothers (compared to multiparous women) to recommend their hospital to friends and family, despite slightly more negative ratings of the hospital and care received while in hospital. Multiparous women have one or more comparison points and have had the opportunity to develop specific expectations that may influence their recommendations [4]. Women having a subsequent birth also may be considering multiple factors when choosing a hospital and be more aware of the influence of health, proximity, facilities and staff on such a decision. There is also potential that first-time mothers are likely to value the only care they have received and, as a form of post-hoc rationalization, are reinforcing for themselves that they made the ‘right choice’ [13]. Overall, two-thirds of women in this sample would recommend the hospital to friends and family compared to 93% of new mothers in Queensland [6]. More detailed analysis of responses and comparison of maternity care from these settings may provide insight into the seemingly low likelihood of NSW women recommending their birth hospital to others.

Differences in responses between women giving birth in metropolitan and rural hospitals were notable. While women in metropolitan hospitals were more likely to positively rate their birth hospital and recommend it to others than their rural counterparts, women in rural hospitals tended to rate staff and care received more highly than women in metropolitan hospitals. Few other Australian studies have examined patient experience by rurality. Miller and colleagues found no difference by area of residence (major city, regional, remote) in perceptions of how well women felt they were looked after during labour and birth [6]. A South Australian analysis found that women who gave birth at rural hospitals had significantly higher overall satisfaction levels than those who gave birth in metropolitan hospitals [8]. It may be in our study that women are separating the care provided by an institution from that provided by individual staff members. In interviews with women receiving maternity care, Jenkins found that criticisms of availability of staff time to spend with patients tended to be described as short-falls of the systems of maternity care rather than individual staff members [22]. It may be for women in rural settings removed from their own environment, friends and family, that relationships with staff become even more important or that staff are personally known by patients. There is some evidence of less access to continuity of carer in rural settings [6] that may make the relationships women develop with each staff member even more important to how they feel about their overall experience of maternity care. In our study, women reporting that they had one doctor in charge were more likely to rate the birth hospital, care and staff more highly than those perceiving more than one doctor (or no doctor) was in charge of their care, however this is likely to be confounded by differences in staffing and models of care in rural and metropolitan settings as well as pregnancy complications.

Two-thirds of women felt they had sufficient information about feeding their baby and caring for their baby. This is lower than 92% of Canadian women receiving sufficient information about infant feeding [10] and the 77-79% of UK women reporting receipt of consistent advice, practical help and active support and encouragement about infant feeding [7]. Comparable Australian data are not available. Multiparous women in our study rated information received about feeding and caring for their baby more positively than first-time mothers. It is quite likely that this reflects reduced information needs in this subgroup of women. Similarly, women with good health status and one doctor perceived to be in charge of their care may reflect a reduced requirement for information; it may be that women with multiple doctors involved in their care are experiencing more complicated pregnancies that by nature may raise questions. Restriction of responses to a rating scale does not allow further exploration of this hypothesis.

In order to explore continuity of care, we examined whether women perceived one doctor or multiple doctors were in charge of their care. While women’s responses were considered according to whether they perceived one doctor to be in charge of their care, it is difficult to interpret results of obstetric patients due to the multiple models of obstetric care provision in New South Wales (e.g. group midwifery practice, obstetrician only, midwifery care for low risk and specialist involvement for higher risk). Perception of one or more doctors in charge of care is likely a poor proxy of continuity of care and does not address midwifery models of continuous care. With changes in maternity services provision over the period of the study, the care received by women has changed. Similar patterns of responses were evident when the 2007–2009 responses were compared to 2010–2011, however small numbers precluded in-depth trend analyses. Changes in maternity care provision are potentially more likely to be identified in dedicated maternity surveys requesting specific information on models of care and experience of specific interventions.

There is an issue around the utility of general overnight patient surveys compared to dedicated maternity surveys for exploring impact of model of obstetric care. Inclusion of the maternity population in general overnight patient surveys can facilitate comparison of satisfaction among medical specialties, however there are specific aspects of care provision such as midwifery compared to obstetrics involvement, and provision of care in delivery suite compared to postnatal ward that are not captured. It is possible that, while the survey is intended to be a survey of overnight inpatient stays, maternity patients rate their overall interaction with their birthing hospital (which may include antenatal clinic and postnatal visits) and are not necessarily restricting their responses to the few days of their birth stay. Dedicated maternity surveys have demonstrated differing levels of satisfaction with antenatal, birth and postnatal care provision, with the lowest ratings associated with postnatal care provision [6,7].

The sample was representative of the wider NSW obstetric population in terms of age group, parity and mode of birth [20]. For example, in 2009, 43% of women were having their first birth, compared to 45% in this patient sample. While a higher proportion of women in this study were English speaking (82% compared with 76% in NSW), following application of survey weighting this reduced to 77%. This is reassuring in the context of response rates in our study of 36-45%; these are comparable to the response rates (35-90%) reported in other overnight patient and maternity surveys) [5-10]. Other strengths of this study include the distribution of surveys by mail which is likely to have resulted in less inhibited responses than if the survey had been distributed in hospital. There are likely to have been some changes to maternity care over the period of the study, however initial analysis of two cohorts (2007–2009, 2010–2011) showed sufficient similarities in responses for the results to be aggregated. Limitations include that while we have a sample representative of women giving birth in hospital and remaining in hospital for at least one night , we cannot know if responses would be the same if we had sampled the wider obstetric population, or at different times during their birth experience. Sourcing responses from an overnight patient survey meant we were unable to compare satisfaction in the antenatal, birth and postnatal period and compare relative satisfaction at each of these time points with those obtained by dedicated maternity surveys. It would have been worthwhile to compare satisfaction by length of stay however this information was not available. Some questions (e.g. perception of one doctor in charge, understandable answers from doctor) are likely to yield different responses among obstetric patients than those from all overnight patients given unique aspects of maternity care including multiple models of care and care providers. High proportions of missing responses and lack of detail on non-English speaking participants precluded analysis of satisfaction among this sub-group. There are recognised limitations of patient experience surveys including the tendency to value care received, lack of experience of other options and the tendency to be more critical of care in a survey than other forms of enquiry [13,23].

## Conclusion

The overall picture of maternity care satisfaction in New South Wales is a positive one, with three quarters of women satisfied with care. This is an important message in the context of an increasing birth rate that has stretched maternity resources in New South Wales [24]. The differences in care ratings among some subgroups of women (for instance, by parity and rurality) may assist in targeting allocation of resources to improve maternity satisfaction. Results from these analyses suggest current policy strategies [1] that optimise the time staff have to get to know their patients (information recording at the bedside, continuity of care) are likely to translate into increased satisfaction. Further resources could be dedicated to ensuring consistency and amount of information provided, particularly to first-time mothers.

### Availability of supporting data

Data were collected by the NSW Ministry of Health which remains the data custodian for these data. The authors do not have permission to release these data.

## Additional file

Additional file 1:
**Questions selected for analysis, NSW Health Hospital Care – Overnight Patient Survey 2007-2011.**

## References

[1] NSW Health Department (2010). Towards Normal Birth.

[2] United Kingdom Royal College of Midwives (2008). Position statement: woman-centred care.

[3] National Health and Medical Research Council (2010). National Guidance on Collaborative Maternity Care.

[4] van Teijlingen E, Hundley V, Rennie A-M, Graham W, Fitzmaurice A (2003). Maternity satisfaction studies and their limitations: ‘what is, must still be best’. Birth.

[5] Brown S, Lumley J (1994). Satisfaction with care in labor and birth: a survey of 790 Australian women. Birth.

[6] Miller Y, Thompson R, Porter J, Prosser S (2011). Findings from the Having a Baby in Queensland Survey, 2010.

[7] Redshaw M, Heikkila K (2010). Delivered with care: a national survey of women’s experience of maternity care 2010.

[8] South Australian Department of Health (2007). Maternity Services in South Australian Public Hospitals: Patient Satisfaction Survey Report.

[9] Rodne T, Daly A (2007). Measuring patient satisfaction in Western Australia, overview of 2005–2006 survey results..

[10] Chalmers B, Dzakpasu S, Heaman M, Kaczorowski J (2008). The Canadian maternity experiences survey: an overview of findings. J Obstet Gynaecol Can.

[11] Sitzia J (1999). How valid and reliable are patient satisfaction data? An analysis of 195 studies. Int J Qual Health Care.

[12] Redshaw M (2008). Women as consumers of maternity care: measuring ‘satisfaction’ or ‘dissatisfaction’?. Birth.

[13] Lumley J (1985). Assessing satisfaction with childbirth. Birth.

[14] New South Wales Department of Health (2009). Patient Survey 2009 Statewide Report.

[15] New South Wales Department of Health (2011). Patient Experience Survey.

[16] Jenkinson C, Coulter A, Bruster S (2002). The picker patient experience questionnaire: development and validation using data from in-patient surveys in five countries. Int J Qual Health Care.

[17] Bureau of Health Information.Insights into Care. Patients’ Perspectives on NSW Public Hospitals. Sydney: Bureau of Health Information; 2010.

[18] Haddock CK, Poston WS, Pyle SA, Klesges RC, Vander Weg MW, Peterson A (2006). The validity of self-rated health as a measure of health status among young military personnel: evidence from a cross-sectional survey. Health Qual Life Outcomes.

[19] Australian Institute of Health and Welfare (2011). Meteor: online metadata registry.

[20] Centre for Epidemiology and Research New South Wales Department of Health (2011). New South Wales Mothers and Babies 2009.

[21] Collins K, O’Cathain A (2003). The continuum of patient satisfaction—from satisfied to very satisfied. Soc Sci Med.

[22] Jenkins MG, Ford JB, Morris JM, Roberts CL. Women’s expectations and experiences in maternity care - what women highlight as most important: a qualitative study. Women Birth. 2014; in press. 10.1016/j.wombi.2014.03.002 24746379

[23] Hall M (1995). Patient satisfaction or acquiescence?.

[24] Lain SJ, Ford JB, Raynes-Greenow CH, Hadfield RM, Simpson JM, Morris JM (2009). The impact of the baby bonus payment in new south wales: who is having “one for the country”?. Med J Aust.

