# Supplementary material for: Are women birthing in New South Wales hospitals satisfied with their care?
Source: BMC Res Notes. 2015 Mar 28;8:106. doi: 10.1186/s13104-015-1067-2 (PMC4378554; doi:10.1186/s13104-015-1067-2)
Supplement: Additional file 1: — Questions selected for analysis, NSW Health Hospital Care – Overnight Patient Survey 2007-2011. [file 13104_2015_1067_MOESM1_ESM.doc]

| **Question number*** | **Question text and response options** |
| --- | --- |
| **12** | **Was there one particular doctor in charge of your care in the hospital?**  O Yes  O No  O Not sure |
| **13** | **When you had important questions to ask a doctor, did you get answers you could understand?**  O Yes, always  O Yes, somewhat  O No  O Did not have questions |
| **17** | **How would you rate the courtesy of your doctors?**  O Poor  O Fair  O Good  O Very good  O Excellent |
| **19** | **When you had important questions to ask a nurse, did you get answers you could understand?**  O Yes, always  O Yes, sometimes  O No  O Did not have questions |
| **23** | **How would you rate the courtesy of your nurses?**  O Poor  O Fair  O Good  O Very good  O Excellent |
| **28** | **Sometimes in the hospital, one doctor or nurse will say one thing and another will say something quite different. Did this happen to you?**  O Yes, always  O Yes, sometimes  O No |
| **41** | **Did you feel you had a care provider who had full understanding of your condition and treatment?**  O Yes, always  O Yes, sometimes  O No |
| **59** | **Did you have a vaginal delivery or a Caesarean section?**  O Vaginal  O Caesarean Section |
| **65** | **Did you get enough information about feeding your baby?**  O Yes, completely  O Yes, somewhat  O No |
| **66** | **Did you get enough information about caring for your baby?**  O Yes, completely  O Yes, somewhat  O No |
| **67** | **Was this your first childbirth experience?**  O Yes  O No |
| **75** | **How would you rate how well the doctors and nurses worked together?**  O Poor  O Fair  O Good  O Very good  O Excellent |
| **76** | **Overall, how would you rate the care you received at the hospital?**  O Poor  O Fair  O Good  O Very good  O Excellent |
| **77** | **Using any number from 0 to 10 , where 0 is the worst hospital possible and 10 is the best hospital possible, what number would you use to rate this hospital during your stay?**  1 2 3 4 5 6 7 8 9 10  O O O O O O O O O O |
| **78** | **Would you recommend this hospital to your friends and family?**  O Yes, definitely  O Yes, probably  O No |
| **88** | **In general how would you rate your health?**  O Poor  O Fair  O Good  O Very good  O Excellent |
| **94** | **What languages do you normally speak at home?**  O English  O Italian  O Greek  O Spanish  O Croatian  O Serbian  O Arabic  O Cantonese  O Mandarin  O Vietnamese  O Korean  O Other (Please specify) |
| **95** | **Are you male or female?**  O Male  O Female |
| **96** | **To which age group do you (the patient) belong?**  O Up to 9 years  O 10 to 14 years  O 15 to 19 years  O 20 to 29 years  O 30 to 39 years  O 40 to 49 years  O 50 to 59 years  O 60 to 69 years  O 70 to 79 years  O 80 years or older |
